# Crohn’s Disease Initially Presenting With Anterior Sclerouveitis

**DOI:** 10.7759/cureus.35373

**Published:** 2023-02-23

**Authors:** Ashley Stahnke, Paige Gioia, Devesh Kumar, Pinky Jha

**Affiliations:** 1 Internal Medicine, Medical College of Wisconsin, Wauwatosa, USA

**Keywords:** inflammatory bowel disease, scleritis and uveitis, sclerouveitis, crohns, clinical case report

## Abstract

This report examines the case of a 32-year-old male who initially presented with symptoms of eye pain, redness, and vision changes, and was subsequently diagnosed with anterior sclerouveitis. One week after his initial visit, the patient presented to the emergency department (ED) with daily bloody stools and left lower quadrant (LLQ) pain. Further workup and examination revealed a diagnosis of Crohn’s disease. This report expands on ocular manifestations of Crohn’s disease and touches on the importance of early gastrointestinal examination in patients who present with ocular symptoms.

## Introduction

Crohn’s disease is an inflammatory bowel disease (IBD), an autoimmune pathology, that most often occurs in patients between the ages of 15 and 35. It can affect the entire gastrointestinal tract from the mouth to the anus, however, it predominately affects the ileum and the proximal colon. In contrast to ulcerative colitis (UC), Crohn’s is characterized by non-continuous lesions, referred to as skip lesions. The most common clinical sequelae include weakness, fatigue, long-term diarrhea with abdominal pain, and variations in weight [1]. Patients have also been found to have ulcers in the mouth and gums, chronic infections of the esophagus with pain, and severe swallowing disorders [2]. Although rare, extraintestinal symptoms include arthropathy, dermatological findings, autoimmune eye pathologies, primary sclerosing cholangitis, amyloidosis, nephrolithiasis, metabolic bone disease, and pulmonary manifestations [3].

We encountered a case of a 32-year-old male who initially presented with anterior sclerouveitis prior to returning one week later with bloody stool; he was subsequently diagnosed with Crohn’s disease. Here we describe the details of this case, discuss possible ocular presentations of Crohn’s disease, and stress the importance of gastrointestinal examination in patients who present with ocular symptoms.

## Case presentation

**Figure 1:**
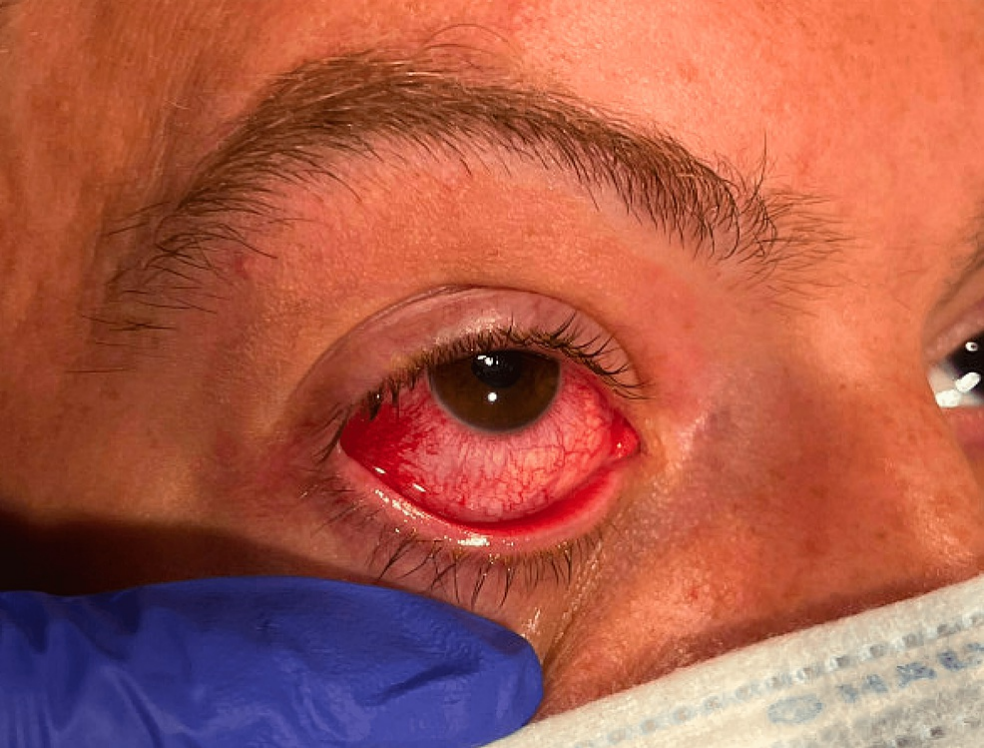
Injected conjunctiva The patient's physical examination demonstrated injected conjunctiva.

**Figure 2:**
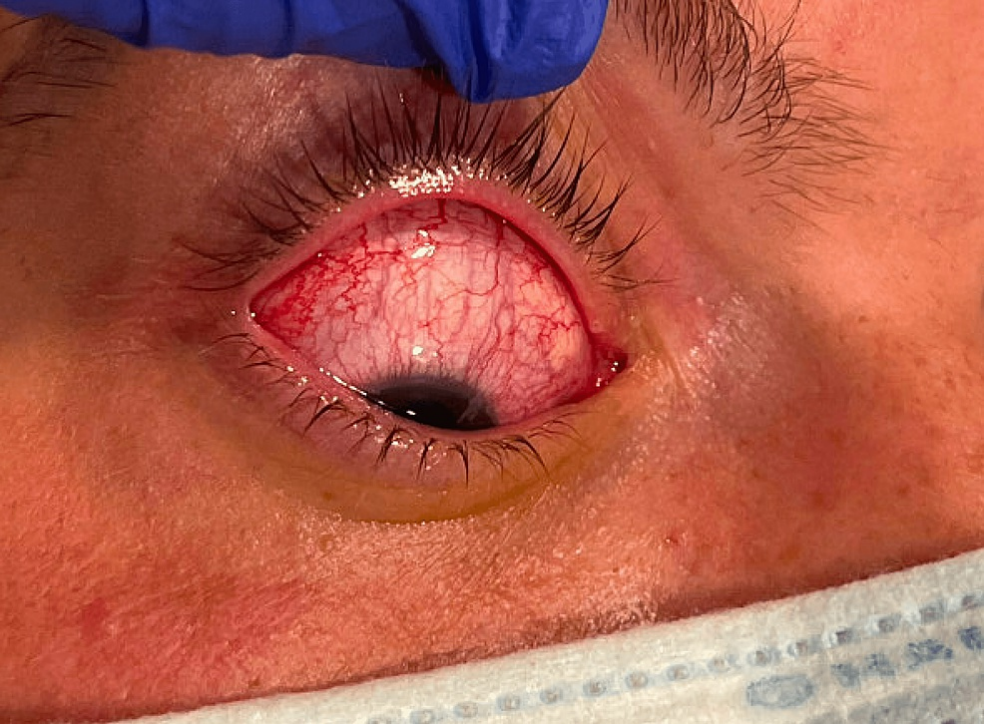
Injected conjunctiva The patient's physical examination demonstrated injected conjunctiva.

A 32-year-old male presented to the emergency department (ED) with right eye pain and redness for four days. The patient’s symptoms included vision changes that were noted as clouding and “small blurry circles”. Achy pain within the eye and progressive redness were also reported. On physical examination, the conjunctiva was injected with normal intraocular pressure, as shown in Figure 1 and Figure 2.

No corneal abrasions or ulcerations were appreciated on the fluorescein lamp. The patient’s history and physical examination prompted a consultation with the ophthalmology team.

Upon examination, ophthalmology administered phenylephrine dilator eye drops, without resolution. Photosensitivity along with an inflamed anterior segment and tenderness to the globe of the eye were appreciated. The following labs were performed and found to be negative: quantiferon, rapid plasma reagin (RPR), antinuclear antibody (ANA), human leukocyte antigen-B27 (HLA-B27), rheumatoid factor (RF), cyclic citrullinated peptide (CCP), and complement. C-reactive protein (CRP) was elevated at 7.65 mg/dL. This subsequently led to the diagnosis of right eye anterior sclerouveitis and the patient was started on cyclopentolate 1% TID, prednisolone acetate 1% every two hours, and naproxen 500mg BID. The patient was discharged home the same day with instructions to follow up with optometry in one week.

**Figure 3:**
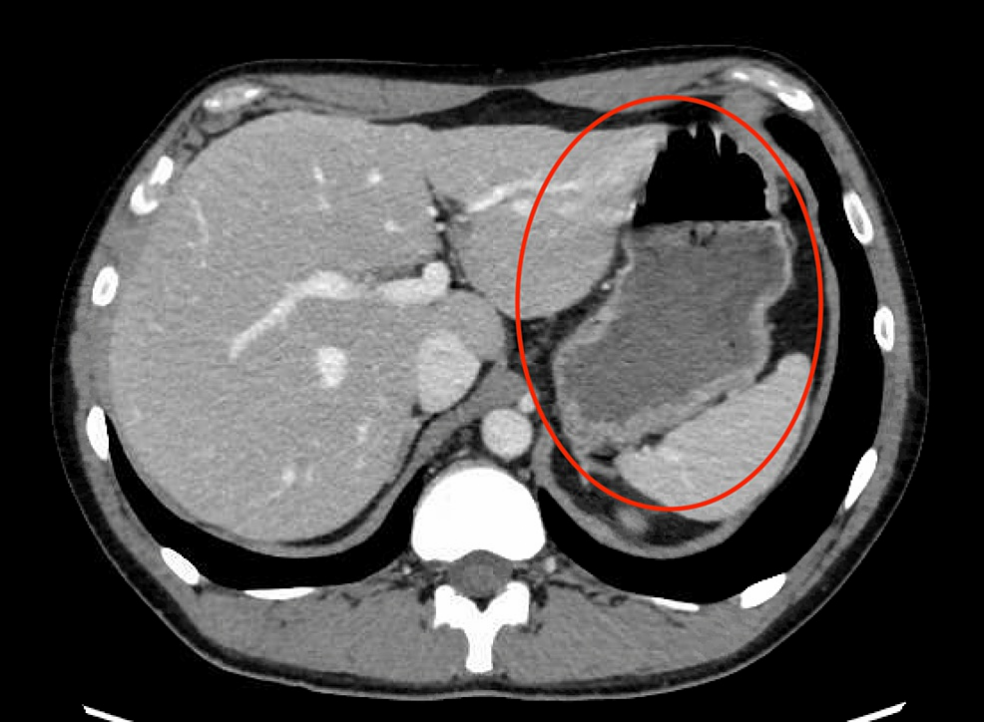
Nonspecific fluid in the left colon An abdominal/pelvis computed tomography (CT) with contrast revealed nonspecific fluid in the left colon.

One week after his initial visit, the patient returned to the ED with complaints of bloody stools. Bloody stools had started a month prior, subsided, and returned daily after his previous ED visit for ocular concerns. The bowel movements contained bright red and maroon blood with occasional left lower quadrant (LLQ) abdominal pain. On presentation, the patient was found to have a low hemoglobin of 10.2 g/dL and CRP continued to be elevated from baseline at 1.69 mg/dL. Physical examination revealed a soft, non-distended abdomen with LLQ and right lower quadrant (RLQ) tenderness to palpation. The rest of the physical exam was grossly normal with exception of his continued erythematous sclera of the right eye. An abdominal/pelvis computed tomography (CT) with contrast revealed nonspecific fluid in the left colon consistent with enteritis, as shown in Figure 3.

The patient was admitted for further workup of his hematochezia and abdominal pain while being started on pantoprazole 40mg BID.

During his hospitalization, the patient continued to have episodes of hematochezia, abdominal discomfort, and cloudy vision in the right eye. The patient’s diagnoses of enteritis and acute sclerouveitis raised concern for an autoimmune etiology.

**Figure 4:**
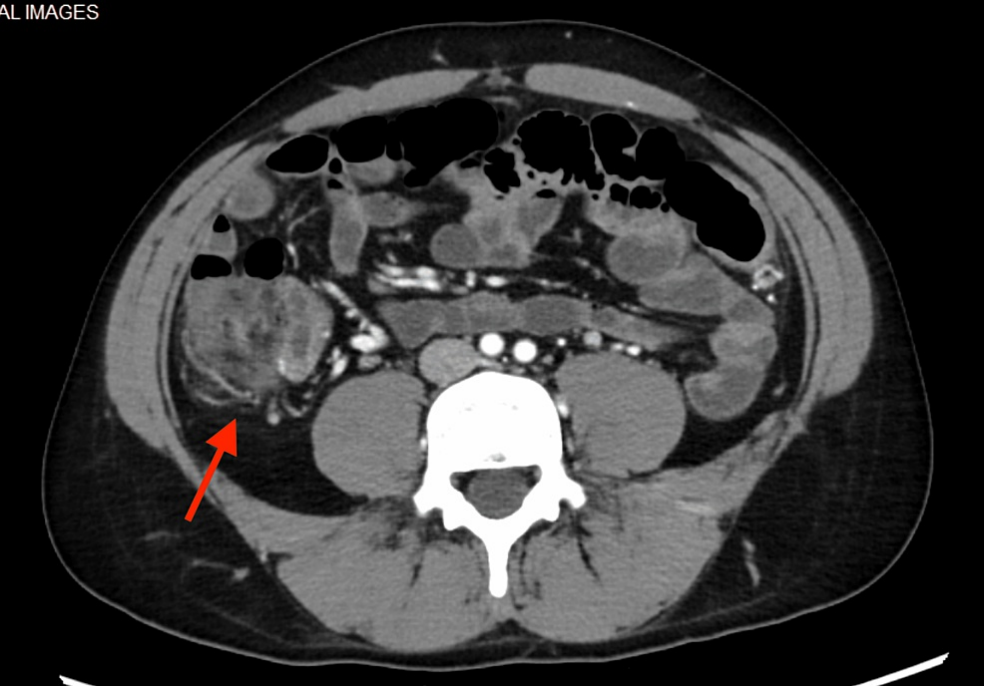
Luminal hyperemia of terminal ileum A computed tomography (CT) enterography with contrast revealed mild luminal hyperemia of the terminal ileum.

A colonoscopy revealed areas of skip ulcerations and cobblestoning throughout the colon along with ulcerations in the transverse ileum, findings most consistent with Crohn’s disease. A CT enterography with contrast also revealed mild luminal hyperemia of the terminal ileum, as shown in Figure 4.

A comprehensive infectious workup was performed and was negative. Biopsies taken during the colonoscopy revealed mildly active enteritis in the terminal ileum, active colitis with ulceration in the ascending colon, and chronic, moderately active colitis with ulceration in the descending colon; these findings confirmed the diagnosis of ileo-colonic Crohn’s disease.

Intravenous (IV) solumedrol 20mg TID was added to the patient's care regimen on hospital day (HD) three; the patient's hematochezia resolved on the same day. After three days, solumedrol was stopped and the patient was transitioned to 40mg of oral prednisone daily. The patient was discharged on HD seven with instructions to follow up with a gastroenterologist (GI) in their IBD clinic to determine a further treatment plan. Upon discharge, the patient had demonstrated no hematochezia for over 72 hours and continued to have active, but improving, conjunctival and scleral injections of the right eye. The patient was instructed to continue taking prednisone 40 mg daily until follow-up.

At his outpatient follow-up with GI, the patient was started on adalimumab to help control his symptoms. The patient has been doing well since starting this medication.

## Discussion

The prevalence of Crohn’s disease has been increasing over the years with most cases arising in patients between the ages of 15 and 35. Since the underlying pathophysiology of Crohn’s involves autoimmune inflammation of the gastrointestinal tract, this disease most often presents chronic diarrhea. However, not all patients with Crohn’s will exhibit this symptom. Additional hallmark symptoms of this diagnosis include abdominal pain (often localized to the RLQ), fatigue, weight loss, fever, growth failure, anemia, and recurrent fistulas [4]. In contrast to UC, rectal bleeding is less common in Crohn’s disease, but it can be found in more severe cases of this diagnosis [1].

Up to 70% of patients with IBD can also exhibit extraintestinal symptoms that affect the joints, skin, kidneys, lungs, hepatobiliary system, and eyes. Risk factors for extraintestinal manifestations include being younger than 40 years old and the female sex [5]. Extraintestinal manifestations can occur in up to one-third of patients with IBD; ocular complications tend to occur in less than 10% of all cases. Ocular complaints can often be nonspecific and easily misdiagnosed, making the evaluation of the eye a critical routine component for patients with IBD. These ocular symptoms, including blurred vision, teary/burning/itching eyes, ocular pain, and conjunctival hyperemia, can precede a diagnosis of Crohn’s disease [6].

While the exact pathogenesis of ocular manifestations due to Crohn’s disease is unknown, one theory is that ocular inflammation is caused by a type III (immune complex-mediated) hypersensitivity reaction to a colonic antigen [7]. This theory explains why patients with colitis or ileocolitis may be more likely to have ocular manifestations than patients with purely small bowel involvement. Of note, ocular involvement tends to be more common in patients with Crohn’s disease (compared to UC) and in the presence of other extraintestinal manifestations.

Studies have found that ophthalmic inflammatory disorders can occur in 3.5%-6.8% of patients with Crohn’s disease, with uveitis occurring in approximately 0.5%-3% of patients with IBD [5,8]. Uveitis is an inflammation of the uveal tract, the middle layer of the eye which provides blood flow to the ocular tissues. In patients with IBD, anterior uveitis typically has an insidious onset, is longstanding and bilateral, and is not related to intestinal disease activity [8].

The course of uveitis does not parallel the activity of IBD, with one study finding that 59% of patients with both conditions were diagnosed with uveitis first [8]. It is essential to recognize the correlation between uveitis and IBD as serious and rare cases may lead to blindness if management is delayed, with uveitis being the third leading cause of irreversible blindness in developed countries [5]. Treatment typically consists of topical steroids and cycloplegics.

Scleritis is an inflammation of the sclera that causes ocular pain which can radiate to the face, periorbital region, and scalp. It is typically worse at night and severe cases can lead to a permanent loss of vision. Scleritis is much rarer in IBD, occurring in less than 1% of cases, but it is extremely important to identify due to its potential for vision loss. It is more common to have involvement of the anterior portion of the sclera in cases of IBD. Systemic treatment is necessary in all cases of scleritis, typically with the use of oral non-steroidal anti-inflammatory drugs. It is also essential to treat the underlying bowel disease, as active bowel disease can lead to the recurrence of ocular manifestations [5].

This case report aims to highlight a rare ocular manifestation of IBD presenting prior to GI symptoms: anterior sclerouveitis. It also aims to encourage clinicians to do a full history and physical examination to identify risk factors and provide the most efficient and compassionate patient care. Early diagnoses, and thus early intervention, of these autoimmune pathologies, are crucial for better long-term outcomes. These include better quality of life, restored vision, and prevention of recurrent ocular manifestations.

## Conclusions

While this patient secured his diagnosis of Crohn's disease relatively quickly after the onset of the initial symptoms, it still ultimately required two ED visits and an admission to achieve. This case highlights how awareness of the extraintestinal manifestations of IBD, along with a full history and physical examination to identify risk factors for Crohn’s disease, could lead to earlier diagnosis. Early diagnosis allows for intervention, which is crucial for better long-term outcomes, including better quality of life and restored vision.
